# Successful removal of intrathyroidal parathyroid adenoma diagnosed and accurately located preoperatively by parathyroid scintigraphy (SPECT-CT)

**DOI:** 10.4103/0972-3919.72689

**Published:** 2010

**Authors:** Dinesh Kumar Kaushal, Atul Mishra, Naveen Mittal, Jayanta K Bordoloi

**Affiliations:** Department of Nuclear Medicine, HERO DMC Heart Institute, Dayanand Medical College, Ludhiana – 141001, Punjab, India

**Keywords:** Intrathyroidal parathyroid adenoma

## Abstract

We describe the case of a large intrathyroidal parathyroid adenoma in a 46-year-old woman who had a history of recently diagnosed hypercalcaemia and a 2-year history of an asymptomatic enlargement of the right lobe of the thyroid. This rare case highlights the potential difficulties that can arise in the evaluation of hyperparathyroidism, especially in cases of multinodular goiter. In some cases, including this one, even a thorough preoperative evaluation that includes radiological studies (ultrasonography and computed tomography [CT]) may not allow for a definitive preoperative diagnosis due to limited sensitivity, especially in multinodular goiter. The overlapping histological features between thyroid and parathyroid lesions can also be problematic at the time of the intraoperative frozen-section evaluation. We present a case in which, with parathyroid scintigraphy and combination of structural and functional imaging (SPECT-CT), we could accurately locate the intrathyroidal parathyroid adenoma in a patient with multinodular goiter.

## INTRODUCTION

The reported prevalence of intrathyroidal parathyroid adenoma varied from 1.4 to 6%.[1,2] Embryologically, parathyroid glands originate from the third and fourth bronchial pouches and migrate caudally to their final positions. It is conceivable that aberrations during migration result in anomalous locations and the parathyroid is trapped within the thyroid as the lateral lobe of the thyroid fuses with the isthmus.[3]

Despite the high success rate achieved at neck exploration by experienced endocrine surgeons, more accurate preoperative localization and intraoperative guidance are required to enable selective minimal surgery and to reduce the operative failure rate.[4,5] Moreover, repeated exploration is associated with a higher rate of complications, including recurrent laryngeal nerve paralysis and hypoparathyroidism.[6]

Anatomic imaging modalities including ultrasonography, computed tomography (CT) and magnetic resonance imaging have a relatively low sensitivity for the detection of parathyroid adenomas.[7,8]

This case supports the use of preoperative SPECT before the initial operation, not only to select patients who are candidates for minimally invasive radioguided surgery[9] but also to provide accurate 3D information on deeply seated or ectopic adenomas.

## CASE REPORT

A 46-year-old old lady presented with newly diagnosed hypercalcemia and elevated parathyroid hormone levels came to our department for a parathyroid scan.

Calcium-14
Phosphorus: 2.6 (2.4–4.5)
Alkaline phosphatase level: 46 (35–104)
PTH: 138 pg/ml (15–65)
Vitamin D: 27.55 ng/ml (20–40)
Renal function tests: Normal
Ultrasonography (USG): MNG. ? Inferior lobulation of enlarged right lobe of thyroid. ??Parathyroid adenoma
CT: Multinodular goiter

**Figure 1:**
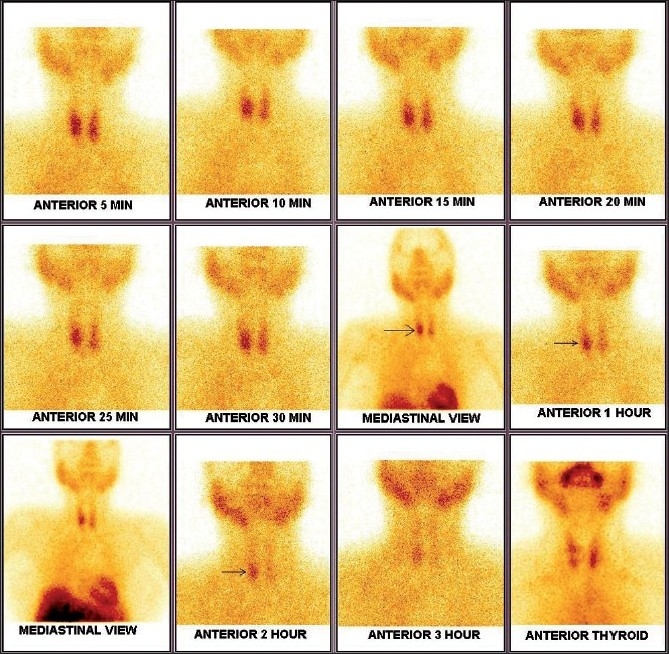
Delayed washout pattern seen involving the lower pole of the right lobe of the thyroid

**Figure 2:**
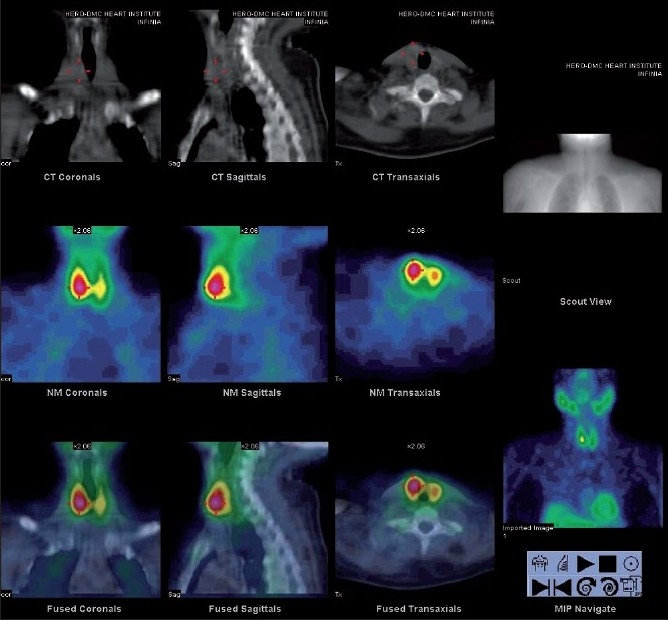
Intrathyroidal parathyroid adenoma in 46 years old woman of primary hyperparathyroidism. Coronal, sagittal and axial delayed-phase fused SPECT/CT images depict a focus of delayed washout at the level of the lower pole of the right thyroid lobe

Parathyroid scintigraphy with SPECT-CT: Anterior planar images of the neck and chest were acquired for 30 min, at 10 min and 120 min after the intravenous injection of 740 MBq 99mTc-MIBI, using a large-field-of-view gamma camera equipped with a parallel-hole collimator. Immediately after the first planar image, a SPECT study was acquired using 60 projections of 30 s each over a 180° anterior arc from the right lateral to the left lateral position in a 128×128 matrix at 3° angular steps. Transaxial, coronal and sagittal slices 1-pixel-thick were reconstructed using a third-order Metz filter set to 8-mm full width at half maximum. Figure 1 shows an abnormal 99mTc-MIBI uptake in the region of the lower pole of the right lobe of the thyroid. Figure 2 illustrates the localization of the abnormal 99m-Tc MIBI uptake in the same patient to be intrathyroidal.

The patient elected to undergo surgery. The right side was investigated first, demonstrating no orthotopic normal or adenomatous inferior parathyroid gland and a normal orthotopic superior parathyroid gland. Palpation of the inferior pole of the thyroid confirmed a nodule, potentially representing intrathyroidal parathyroid adenoma, embedded in the thyroid tissue. The patient underwent right lobectomy

Postop biopsy: Parathyroid adenoma
Postop calcium: Normal, 10.3
The postoperative course was uneventful.

## DISCUSSION

We suggest, even in patients with coexistent primary hyperparathyroidism and multinodular goiter, that if radiotracer localizes to a particular nodule on scintigraphy sparing all other nodules, shows delayed washout pattern, no other site of ectopic parathyroid tissue is found and USG findings are suspicious for parathyroid adenoma, combined parathyroid scintigraphy (SPECT-CT) and USG is effective in suggesting a preoperative diagnosis of intrathyroidal parathyroid adenoma. SPECT-CT accurately located the intrathyroidal location of the parathyroid adenoma in this case. Despite its rarity, the possibility of an intrathyroidal parathyroid should be kept in mind, and, when meticulous bilateral exploration of the neck fails to identity the hyperfunctioning gland, the surgeon should consider hemithyroidectomy/lobectomy based on preoperative USG and parathyroid scintigraphy (SPECT-CT). In patients with multinodular goiter, ultrasound has limited sensitivity and any abnormal 99m-Tc MIBI concentration should not be ignored as a false-positive concentration in a thyroid nodule and should be thoroughly evaluated.
